# Identification, Selection and Immune Assessment of Liver Stage CD8 T Cell Epitopes From *Plasmodium falciparum*

**DOI:** 10.3389/fimmu.2021.684116

**Published:** 2021-05-07

**Authors:** Kenneth D. Tucker, Brian C. Schanen, Timothy W. Phares, Emily Sassano, Frances E. Terry, Pooja Hindocha, Leonard Moise, Vinayaka Kotraiah, William D. Martin, Anne S. De Groot, Donald R. Drake, Gabriel M. Gutierrez, Amy R. Noe

**Affiliations:** ^1^ Leidos Life Sciences, Leidos Inc., Frederick, MD, United States; ^2^ Sanofi Pasteur, Orlando, FL, United States; ^3^ EpiVax Inc., Providence, RI, United States; ^4^ University of Georgia Center for Vaccines and Immunology, Athens, GA, United States

**Keywords:** malaria, vaccine, epitopes, CSP, SPECT-2, MIMIC

## Abstract

Immunization with radiation-attenuated sporozoites (RAS) has been shown to protect against malaria infection, primarily through CD8 T cell responses, but protection is limited based on parasite strain. Therefore, while CD8 T cells are an ideal effector population target for liver stage malaria vaccine development strategies, such strategies must incorporate conserved epitopes that cover a large range of class I human leukocyte antigen (HLA) supertypes to elicit cross-strain immunity across the target population. This approach requires identifying and characterizing a wide range of CD8 T cell epitopes for incorporation into a vaccine such that coverage across a large range of class I HLA alleles is attained. Accordingly, we devised an experimental framework to identify CD8 T cell epitopes from novel and minimally characterized antigens found at the pre-erythrocytic stage of parasite development. Through in silico analysis we selected conserved *P. falciparum* proteins, using *P. vivax* orthologues to establish stringent conservation parameters, predicted to have a high number of T cell epitopes across a set of six class I HLA alleles representative of major supertypes. Using the decision framework, five proteins were selected based on the density and number of predicted epitopes. Selected epitopes were synthesized as peptides and evaluated for binding to the class I HLA alleles *in vitro* to verify in silico binding predictions, and subsequently for stimulation of human T cells using the Modular IMmune In-vitro Construct (MIMIC^®^) technology to verify immunogenicity. By combining the in silico tools with the *ex vivo* high throughput MIMIC platform, we identified 15 novel CD8 T cell epitopes capable of stimulating an immune response in alleles across the class I HLA panel. We recommend these epitopes should be evaluated in appropriate *in vivo* humanized immune system models to determine their protective efficacy for potential inclusion in future vaccines.

## Introduction

Immunization with radiation-attenuated *Plasmodium* sporozoites (RAS) provides protection from malarial infection in mice, non-human primates, and humans (1–5). The irradiated sporozoites invade the liver, however their division is arrested and infection cannot progress to the blood stage of malaria (6, 7). This sterile immunity requires a CD8 T cell response targeting pre-erythrocytic stage antigens (8–12). The discovery that RAS immunization can confer sterile immunity led to a focus on discovering novel liver stage antigens, as many of the antigens responsible for conferring protection are unknown (13–15).

An obstacle in developing a T cell-focused vaccine is the heterogeneity of human leukocyte antigen (HLA) in the human population (16). Although some epitopes bind class I HLA alleles across different supertypes, due to HLA restriction, vaccines that rely on CD8 T cell responses are likely to require multiple epitopes capable of binding (and being presented by) different class I HLA alleles ensuring broad HLA coverage and provide protective immunity across diverse populations (16, 17). HLA alleles can be clustered into supertypes based upon their ability to bind overlapping sets of peptides with related amino acid sequences, which allows reduction in the number of epitopes while still covering immunity to a broad segment of the population (18, 19). Computational vaccinology analyses typically utilize one HLA allele from each set of HLA supertypes to facilitate analysis whilst generating epitope sets capable of binding to a large section of the world-wide population. For this analysis we used the following six class I supertype HLA alleles, A*0101, A*0201, A*0301, A*2402, B*0702, and B*4403, to achieve coverage of over 90% of the human population (20). On the basis of HLA genotypic frequencies, a set of epitopes restricted by these six supertypes (and the related allele family members they represent) is predicted to cover 96.5% of the population in North Africa and 92.6% of the population in sub-Saharan Africa (21).

To address impacts of HLA restriction, vaccines must either be based on a target protein containing a large number of CD8 T cell epitopes (i.e., a protein target with a high epitope content) or incorporate T cell epitopes from multiple protein targets to provide sufficient HLA coverage. With regard to the latter strategy, two high-profile vaccines have been developed: (1) a moderately successful vaccine developed for malaria that includes the Thrombospondin-Related Anonymous Protein (TRAP) antigen as well as a multi-epitope string containing CD8 and CD4 T cell epitopes from TRAP, circumsporozoite protein (CSP), export protein 1 (EXP-1), liver stage antigen-1 (LSA-1), liver stage antigen-3 (LSA-3); and sporozoite threonine-asparagine-rich protein (STARP), which is referred to as multi-epitope thrombospondin-related adhesion protein (ME-TRAP) (22), and (2) a relatively unsuccessful multivalent DNA vaccine (identified as EP-1300) composed of 38 CD8 and 16 CD4 T cell epitopes derived from TRAP, CSP, EXP-1 and LSA-1 expressed as a single protein (23).

New malaria vaccines are needed. While RAS provides protective immunity against homologous challenge, protection against heterologous challenge has proven problematic (24). In addition, the requirements for a cold chain to maintain the infective irradiated sporozoites and need for repeated immunizations pose significant obstacles to its wide-spread use (25, 26). Both CSP and TRAP subunit-based vaccines (RTS,S and ME-TRAP, respectively) have successfully elicited sterile immunity against malaria infection in humans; however, both have demonstrated only modest efficacy (27). The protection elicited using subunit-based vaccines is low compared to the protection seen with RAS, suggesting the key protective antigens have not been identified and/or the highly protective RAS immunity is generated in response to multiple antigens expressed in the liver stage (16, 27–29). In either case, discovery of novel antigens and their inclusion in multi-antigen/subunit vaccines is needed to increase effectiveness of a liver stage malaria vaccine.

In response to the need for validating novel antigens and identifying functional T cell epitopes, we developed and applied an experimental framework to identity novel vaccine targets from the large set of *P. falciparum* proteins previously shown to be expressed in the late liver stage and into the blood stage of malaria. To this end, we leveraged previous studies to identify a panel of 100 proteins to serve as the initial sequence inputs for in silico T cell epitope analyses. Through an iterative process based on predicted immune potential, five proteins were selected for more intensive in silico T cell epitope analyses to identify a set of epitopes with high conservation and high prediction scores for class I HLA binding. The resulting predicted epitope sets were synthesized as peptides and assessed *in vitro* to verify HLA allele binding and *ex vivo*, for the ability to stimulate cytokine responses using the Modular IMmune In-vitro Construct (MIMIC^®^) technology, an *ex vivo* model of human immunity.

## Materials and Methods

### BLAST

BLAST searches were performed using the amino acid sequence derived from *P. falciparum* 3D7, obtained from the PlasmoDB (30, 31). Each protein sequence was compared to *P. vivax* taxid 5855 to evaluate conservation of the protein and provide a score for the identity and coverage of the protein in *P. vivax*. Each protein sequence was compared to human proteins using taxid 9606 to evaluate the identity to proteins found in humans, which could result in immune tolerance. Predicted T cell epitopes were compared to *P. vivax* taxid 126793 to evaluate conservation of the amino acid sequence relative to *P. vivax* strain Salvador-1 (Sal-1). This included evaluating the complete peptide sequence for identity, as well as evaluating only the predicted TCR-interacting amino acids to determine the sequence identity. The Sal-1 sequence is a high fidelity genome sequence that provides reliable amino acid sequences for use in identifying epitopes conserved across a different species of *Plasmodium* that is infective to humans.

### Identification of Potential T Cell Epitopes and Protein Immunogenicity

Epitopes were predicted from each protein using the EpiMatrix tool from the iVAX toolkit (32). Protein sequences from *P. falciparum* 3D7 were utilized. For class I epitope prediction, each input sequence was parsed into all possible 9-mer and 10-mer frames, where each frame overlapped the last by eight or nine amino acids, respectively. Each 9-mer or 10-mer frame was scored for its potential to bind to a panel of six class I HLA alleles (A*0101, A*0201, A*0301, A*2402, B*0702, and B*4403) representing six supertypes. For class II epitope prediction, each input sequence was parsed into all possible 9-mers, where each frame overlapped the previous frame by eight amino acids. Each 9-mer was evaluated for its relative probability to bind to a panel of eight class II HLA alleles (DRB1*0101, DRB1*0301, DRB1*0401, DRB1*0701, DRB1*0801, DRB1*1101, DRB1*1301, and DRB1*1501) representing eight supertypes. Raw EpiMatrix scores were normalized and presented on a “Z” scale. Scores above 1.64 represent the top 5% of the normalized distribution of all EpiMatrix scores. These peptides have a significant probability of binding to the subject’s HLA allele(s). EpiMatrix Z-scores above 2.32 represent the top 1% of predicted binders and are even more likely to bind the subject’s HLA allele(s).

The overall immunogenic potential of each protein was scored according to the EpiMatrix Protein Score and compare against benchmark sequences (32). An Immunogenicity Score is developed based upon the density of predicted epitopes and is normalized for length, allowing for the comparison of proteins of different lengths on a common epitope density scale. Scores range from -50 to greater than 50, with proteins scoring above 20 considered to have significant immunological potential.

### Analysis of ‘Human-Ness’ as a Flag for Possible Immunosuppressive Responses

To identify and exclude peptides less likely to induce effective immune responses, the peptides meeting the top 5% probability to bind class II HLA alleles were analyzed using the JanusMatrix tool from the iVAX tool kit, as previously described (32, 33). In brief, this tool examines human sequence similarity with respect to the HLA and T cell receptor (TCR) face-interacting residues of an epitope (i.e., human-ness) to flag sequences that could potentially elicit immunosuppressive responses due to homology with sequences encoded by the human genome. For example, lower responses in epitopes flagged for human-ness may be found to induce a T regulatory (Treg) cell response, as the Treg repertoire plays a role in the maintenance of self-tolerance (34). Published epitopes with a large number of cross-conserved matches to human sequences for the TCR-interacting residues are more highly correlated with IL-10 than IL-4 cytokine responses and more likely to be capable of inducing immune tolerance [reviewed in (35)]. The JanusMatrix Human Homology Score of a given peptide or protein indicates the number of potential human-ness triggers or flags, with a higher JanusMatrix scores indicating a bias towards immune tolerance (36). The use of JanusMatrix Scores in vaccine development for viral and malaria applications is elaborated in (35) and (36), respectively. Ninety-five percent of randomly generated predicted ligands to HLA-DRB1 alleles have JanusMatrix Scores between zero and two. Therefore, a JanusMatrix Score greater than two is a threshold for higher than typical human-ness flags. For this effort, we used a JanusMatrix Protein Score cut-off of one and a JanusMatrix Epitope Score cut-off of three as exclusion criteria for our decision framework. For the analysis of the 100 selected proteins, a summary JanusMatrix score for each protein was determined using the average JanusMatrix score for all the putative class II epitopes in the input pathogen protein; therefore, a lower cut-off was used.

### Peptide Synthesis

Peptides were produced by 9-fluoronylmethoxycarbonyl (Fmoc) synthesis at 21st Century Biochemicals. For class I studies, the peptides were designed with a C-terminal hydroxyl group cap and quality was ascertained by HPLC, mass spectrometry, and UV scan (ensuring purity, mass, and spectrum, respectively). In all cases, the amino acid content of each peptide was determined to enable reconstitution at highly accurate molarity. For binding assays peptides were used at >85% purity, and for *ex vivo* T cell analysis peptides were used at >95% purity.

### HLA Class I Binding Assay

Binding of the predicted class I epitopes to the target HLA alleles was evaluated by Pure Protein, LLC using the procedure previously described (37). The cell-free class I HLA binding assay allows for *in vitro* quantification of peptide-HLA binding affinity in a competition format. In this assay, a fluorescently labeled, high binding, reference peptide was loaded onto 384-well plates along with unlabeled experimental peptides. The reference peptides were as follows: IADMGHLKY (A*0101), GLMTTVHAI (A*0201), SLFRAVITK (A0301), PYVSRLLGI (A*2402), IPSYKKLIM (B*0702), and MEVDPIGHLY (B*4402). Negative control peptides were IPSYKKLIM (A*0101, A*0201, A*0301, and A*2402) and SLFRAVITK (B*0702 and B*4402). To remove endogenous peptides and render the HLA A and B molecules receptive to binding, HLA alleles were heated at 53°C for 15 minutes and immediately added to the peptides. The mixture was then allowed to stand for three days. Once the mixture had reached steady equilibrium (at 72 hours), displacement of the high binding control peptide was measured through Fluorescence Polarization. Peptides were assayed against a panel of six common class I HLA alleles. They are A*0101, A*0201, A*0301, A*2402, and B*0702 and B*4402. For each experimental peptide, samples were prepared at a high concentration (80,000 nM) and a low concentration (500nM for A*2402 and B*0702, 2,000 nM for remaining alleles). The FITC-labeled control peptide was diluted to 2 nM. An HLA-only and peptide-only preparation anchored the top and bottom, respectively, of a four-condition curve. Binding of experimental peptides was expressed as the percent inhibition of the labeled control peptide (experimental fluorescence/control fluorescence, multiplied by 100). The percent inhibition values for each experimental peptide were used to calculate the concentration which inhibits 50% of the labeled control peptide’s specific binding. This value is referred to as the peptide’s inhibitory constant (IC_50_). Based on a peptide titration with four concentrations (including zero), non-linear regression analysis was performed to fit the data to a curve and an IC_50_ value was calculated.

### MIMIC^®^ CD8 T Cell Stimulation Assay

Peripheral blood mononuclear cells (PBMCs) from healthy donors residing in the United States and enrolled in a Sanofi Pasteur-VaxDesign campus apheresis program (Protocol CRRI 0906009) were collected. Informed consent was obtained from each subject prior to enrollment. All blood samples obtained and used in this study were collected from consenting participants in compliance with the institutional review board (IRB) approved protocol (Protocol CRRI 0906009). Apheresis collections were performed by OneBlood (Orlando, FL) using standard techniques approved by their IRB. Within hours following their harvest from the donor, the enriched leukocytes were centrifuged over a ficoll-plaque PLUS (GE Healthcare, Piscataway, NJ) density gradient. PBMCs at the interface were collected, washed, cryopreserved in IMDM media (Lonza, Walkersville, MD) containing autologous serum and DMSO (Sigma–Aldrich, St. Louis, MO), and stored in vapor phase liquid nitrogen until needed.

Monocytes were purified from total PBMCs by anti-CD14 antibody conjugated magnetic beads (Stemcell Technologies) and cultured for 6 days in serum-free CellGro Dendritic Cell Medium (CellGenix) supplemented with 100 ng/mL GM-CSF (R&D Systems, Minneapolis, MN) and 25 ng/mL IL-4 (R&D Systems). Dendritic cells (DCs) were matured using 10ng/mL of LPS (Sigma–Aldrich) and 100IU/mL of IFNγ (Peprotech). The matured DCs were then harvested for use in the CD8 T cell stimulation assay within 16 hours of maturation.

CD8 T cell stimulation assays were performed using protocols established at Sanofi Pasteur–VaxDesign Campus (38). CD8 T cells were sorted by negative selection using EasySep Human CD8 T cell isolation kit (Stemcell technologies). The resulting CD8 T cells (>95% pure) were plated at a concentration of 3 x 10^6^/mL in X-VIVO 15 media supplemented with 5ng/mL IL-7 (R&D Systems) overnight. The DCs were either untouched (control wells) or pre-pulsed for at least 2 hours with pooled peptides (5 µg/mL per peptide). CD8 T cells were then harvested and co-cultured with autologous DCs at a ratio of 60:1 in X-VIVO 15 media supplemented with 30ng/mL IL-21 (R&D Systems). On day 3 of the co-culture half of the culture media was replaced with fresh X-VIVO 15 media containing IL-7 and IL-15 (R&D Systems) at a final concentration of 5ng/mL. On day 5 the co-culture was transferred from the original 48 well plate to a 12 well plate and 1mL of fresh X-VIVO 15 was added containing IL-7 and IL-15 at a final concentration of 5ng/mL.

After a 12-day incubation period, lymphocytes were harvested and evaluated for effector activity using intracellular cytokine staining (ICCS). For the ICCS, autologous DCs were matured as described above. The matured autologous DCs were left untouched or pulsed separately with individual peptides (5 µg/mL per peptide) overnight. The pre-pulsed DCs were then harvested and cultured with the primed T cells. The co-cultures were allowed to incubate for 7 hours. For the final 5 hours of the restimulation, 1µg/mL brefeldin A (Sigma–Aldrich) was added to prevent protein egress from the Golgi apparatus. Following the incubation period, the cells were labeled with the Live/Dead Fixable Stain Kit (Invitrogen, Carlsbad, CA), treated with cytofix/cytoperm and perm/wash reagents from BD Biosciences (San Jose, CA), and then labeled with Bioscience (San Diego, CA) antibodies specific for human IFNγ, TNF-α, IL-2, and granzyme B. The samples were acquired on an LSRII flow cytometer (BD Biosciences) and analyzed using FlowJo software (TreeStar, Ashland, OR). The stimulation index for the epitope-specific responses was calculated using the following: Stimulation Index = [Individual Peptide Restimulation (Peptide pool culture)]/[No Antigen Restimulation (Peptide pool culture)]. As a positive control for CD8 T cell stimulation the MHC class I control peptide pool containing a total of 32 peptides, each corresponding to a defined HLA class-I restricted T cell epitope from cytomegalovirus, Epstein-Barr virus, and influenza virus [CEF-MHC class I control peptide pool “plus” (Cellular Technology, Ltd.)] was used. CEF-MHC class I peptide pool was used at the manufacturer’s suggested concentration of 2µg/mL.

## Results

### Identification of the Liver Stage Proteins for Analysis and Overview of the Experimental Framework

**Figure 1:**
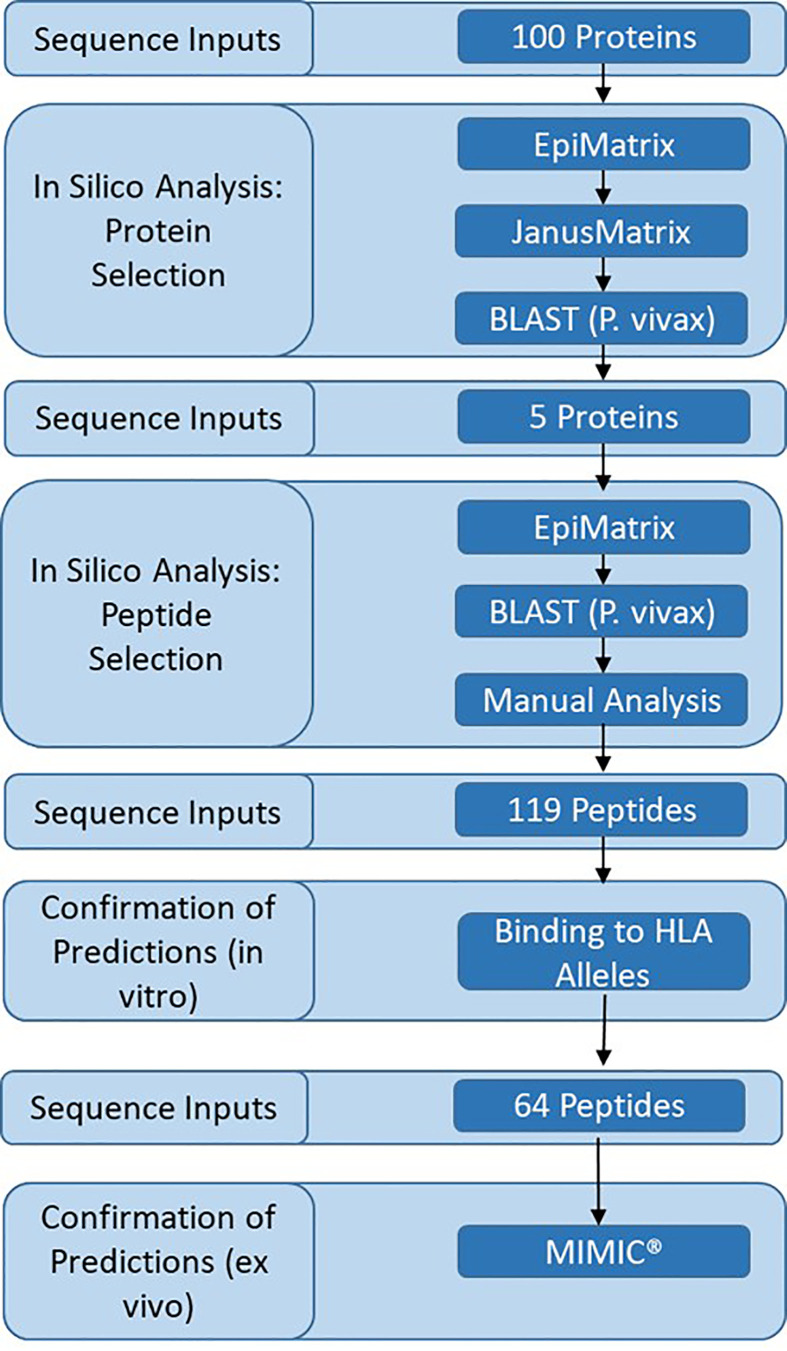
Experimental framework and analysis workflow.

We sought to identify novel proteins and T cell epitopes expressed in the late liver stage of malaria. Analysis was performed on a subset of 100 proteins expressed in the pre-erythrocytic stage of malaria (**Supplementary Table 1**). This subset included proteins previously evaluated for T cell responses in volunteers vaccinated with irradiated sporozoites (14, 39) and as potential subunit vaccines in animal models or clinical trials (40). Also included were thirty-six proteins that were expressed in both the liver stage (41) and blood stage of malaria (42), and were indicated to be conserved and uncharacterized in PlasmoDB (31). Analysis of the 100 proteins was performed in several steps and included a decision framework for selection of relatively novel, conserved proteins that were immunogenic across a diversity of HLA types (**Figure 1**). The framework started with in silico analyses, moved to *in vitro* assessments, and then to *ex vivo* assessments as follows: (1) in silico analysis of *P. falciparum* protein sequences was performed using the EpiMatrix and JanusMatrix tools to determine overall predicted epitope content for each protein input sequence and identify any proteins containing sequences overlapping the human proteome that were flagged for human-ness. This was followed by a protein conservation analysis of the *P. falciparum* input sequences compared to the *P. vivax* orthologs using BLAST, yielding five antigens that went on to (2) in silico analysis using the EpiMatrix and JanusMatrix tools to identify all predicted epitopes within the proteins and identify any predicted epitope containing sequences overlapping the human proteome that were flagged for human-ness, which was followed by an epitope conservation analysis of the *P. falciparum* predicted epitope set compared to the *P. vivax* ortholog sequences using BLAST, yielding peptides that went on to (3) *in vitro* HLA binding evaluations, that yielded peptides for (4) *ex vivo* human PBMC assessments to determine cytokine-producing T cell response profiles in the MIMIC platform.

### *In Silico* Analysis of 100 *P. falciparum* Proteins

To develop new potential antigens for application in a pre-erythrocytic stage vaccine to malaria, we sought novel and minimally characterized proteins that were indicated to support a T cell response across a population with a diversity of HLA types. To identify the proteins with a relative large number of predicted T cell epitopes, the proteins were evaluated using EpiMatrix to determine EpiMatrix Protein Scores for each of the 100 proteins (**Supplementary Table 1**) (32). The EpiMatrix Protein Score increases as the predicted density of T cell epitopes in a protein increases. EpiMatrix Protein Scores are computed for class I or class II predicted epitopes content by summing the top 5% of Z-score outputs for the subject HLA allele set, determining the deviation from random expectation and normalizing the results for a 1000-amino acid protein length. On a scale with random expectation set at zero, EpiMatrix Protein Scores above zero indicate the presence of more MHC ligands than expected due to chance and denote a higher potential for immunogenicity. Scores below zero indicate the presence of fewer potential MHC ligands than expected and a lower potential for immunogenicity. Proteins scoring above 20 on this scale are considered to have significant immunogenic potential. In the current study, proteins with class I immunogenicity scores above 20 were selected for further analyses (**Supplementary Table 1**).

In parallel to the EpiMatrix analysis the proteins were scored for their human-ness using the JanusMatrix tool (**Supplementary Table 1**) (32, 33). The JanusMatrix Protein Score measures the average number of cross-conserved matches to human sequences for the TCR-interacting residues in the set of predicted epitopes for a protein and flags the potential of immunosuppressive responses when predictive epitopes have a larger then typical amount of cross-conservation with the human proteome. As the JanusMatrix Score increases, the potential for an epitope to induce an effective immune response decreases. Proteins with a JanusMatrix Protein Score of less than 1.0 were selected for further analysis.

Proteins were also assessed for *P. vivax* sequence conservation relative to the *P. falciparum* sequence using BLAST. For this analysis the summation of the percentage of the query coverage and the percentage of identity to *P. vivax* was used as the score of relative conservation. Proteins indicated to be highly conserved (% query coverage + % identity to *P. vivax* > 150) were selected for further analysis (**Supplementary Table 1**).

Using the criteria of high EpiMatrix Protein Score and low JanusMatrix Protein Score, to indicate overall immune potential, and relatively high conservation to *P. vivax*, eleven relatively uncharacterized proteins were selected for further assessment (**Table 1**). Of note, while slightly outside the selection parameters, PFC0450w, and PFD0425w were included with the selected proteins as these two proteins were close to meeting all of the selection criteria. Moreover, the literature suggests that PFC0450w and PFD0425w may be associated with protective immunity (14, 39).

**Table 1:** Identity and immunogenicity scores for eleven selected proteins.

Protein	BLAST P. vivax		Class I EpiMatrix Score	JanusMatrix Score
	% Query Coverage	% Identity		
PF08_0081	100	63	23.97	0.76
PF10_0170	97	63	59.84	0.7
PF13_0116	97	62	49.33	0.98
PF14_0105	94	77	74.73	0.81
PF14_0301	100	75	54.39	0.76
PF14_0593	93	76	56.47	0.98
PFC0450w	100	83	103.81	1.15
PFC1055w	100	57	35.77	0.96
PFD0425w	99	57	48.71	1.31
PFD0430c	99	55	28.08	0.92
PFE1025c	100	55	51.98	0.69

### Selection to the Top 5 *P. falciparum* Proteins

A comparative analysis was conducted on the eleven proteins in **Table 1** to determine which had the highest number and density of predicted class I epitopes across the HLA allele panel. The EpiMatrix tool was used to identify the number of class I epitopes that were predicted to bind to at least one of six class I HLA alleles (A*0101, A*0201, A*0301, A*2402, B*0702, B*4403) (**Table 2**). As every HLA allele in the panel represents a large family of closely-related alleles (i.e., supertype), analysis of these six HLA alleles can be used to infer epitope coverage relevant to over 90% of the global human population (20). While the selected proteins in **Table 1** had high EpiMatrix Protein Scores, indicating a large number of class I epitopes, the numbers of predicted epitopes for each HLA allele were not evenly distributed for the different proteins. Particularly, epitopes predicted to bind HLA-B*0702 were generally fewer than for other alleles. Due to the limited number of epitopes for HLA-B*0702, the proteins PF10_0170 and PFC0450w were deprioritized (**Table 2**).

**Table 2:** Number of class I epitopes for each of six selected class I HLA supertype alleles in each of the eleven selected proteins.

Protein	Class I	Number of Significant EpiMatrix Hits						Highest JanusMatrix Score
	EpiMatrix Score	A*0101	A*0201	A*0301	A*2402	B*0702	B*4403	
PF08_0081	23.97	53	34	35	63	21	31	2
PF10_0170	59.84	13	8	5	18	2	12	3
PF13_0116	49.33	114	79	106	149	22	69	7
PF14_0105	74.73	33	21	38	43	5	21	5
PF14_0301	54.39	26	14	27	29	7	22	3
PF14_0593	56.47	134	88	109	135	29	107	3
PFC0450w	103.81	3	19	11	15	3	9	2
PFC1055w	35.77	52	37	63	63	6	49	5
PFD0425w	48.71	109	58	69	95	36	60	6
PFD0430c	28.08	76	51	56	65	20	50	3
PFE1025c	51.98	23	13	30	36	8	21	2

In addition to the predicted class I HLA allele analysis, the eleven proteins in **Table 1** were reevaluated for human-ness flags using the JanusMatrix tool. In the original JanusMatrix analysis of the 100 proteins, the average human cross-conserved score was calculated for the whole protein (i.e., the JanusMatrix Protein Score). As JanusMatrix Protein Scores represent the average scores of all predicted epitopes within a protein, a low score could be due to a large number of low scoring predicted epitopes which, when averaged, can mask a few epitopes with high JanusMatrix scores. Therefore, JanusMatrix analysis was performed to score each predicted class II epitope within the eleven proteins. As anticipated, several epitopes with scores higher than the average score of the whole protein were detected. Consequently, four of the proteins, PF13_0116, PF14_0105, PFC1055w, and PFD0425w, contained predicted epitopes with a JanusMatrix score greater than 3.0 cut-off and were deprioritized (**Table 2**). As a result, five proteins, PF08_0081, PF14_0301, PF14_0593, PFD0430c, and PFE1025c, were advanced as potential vaccine candidates.

### *In Silico* Analysis to Identify and Select Predicted Class I Epitopes

**Figure 2:**
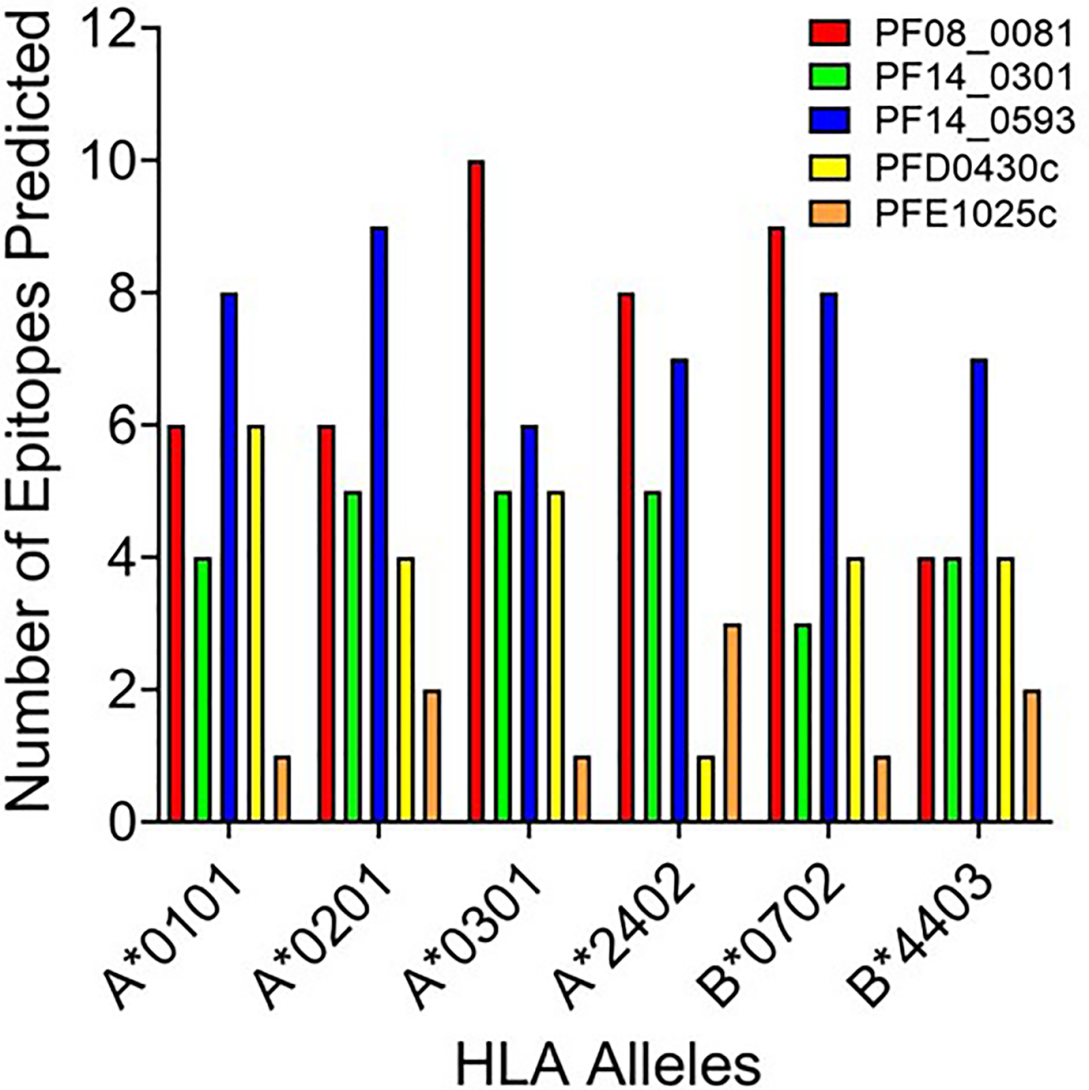
The number of predicted epitopes for each HLA allele supertype from each of the five proteins. Shown are the number of peptides predicted by EpiMatrix to bind a specific class I HLA allele and selected for further development.

Based upon the EpiMatrix T cell epitope analysis, PF08_0081, PF14_0301, PF14_0593, PFD0430c, and PFE1025c collectively contained 1,413 class I predicted epitopes in the top 5% Z-scores. Initial selection from the predicted epitopes was informed by conservation of the epitopes in *Plasmodium*. Using the peptide sequence from *P. falciparum* strain 3D7 the predicted epitopes were evaluated for conservation in *P. vivax* strain Sal-1 using BLAST. Since the proteins sourced for novel T cell epitopes have not been extensively studied, there are limited data of variation in strains of *P. falciparum*. Therefore, conservation of the amino acid sequence of the peptide relative to the orthologous protein in *P. vivax* strain Sal-1 was evaluated using BLAST. This strain of *P. vivax* has a high fidelity genome sequence that is available to the public and provides a reliable predicted amino acid sequence of the orthologous proteins. Predicted epitopes with conserved sequences were preferentially selected. Conservation of sequence and balanced distribution of the number of predicted epitopes across the selected six HLA allele panel were used as criteria to select epitopes for analysis. *P. falciparum* peptide sequences with identity to the *P. vivax* sequence in the orthologous protein were preferentially selected; however, peptides sequences with no identity were also selected to increase the number of predicted epitopes for each allele. The selection criteria for non-conserved epitopes targeted predicted epitopes with the highest Z-score. Selection of peptide sequences targeted an even distribution of the number of sequences predicted to bind to each of the HLA alleles. As the predicted epitopes were selected, the sequences were manually evaluated for the physical properties that could affect solubility and stability. Peptide sequences containing charged amino acids and relatively low hydrophobicity were selected. Also, peptide sequences with amino acid in positions and order that may prove problematic for chemical synthesis (e.g., N-terminal glutamine) or with oxidizable amino acids were excluded. Based on these criteria, 119 predicted class I epitopes were selected for further analysis, of which 98 were predicted to bind only a single class I HLA allele and the remainder were predicted to bind two or more class I HLA alleles (**Supplementary Table 2**). In total, the 119 peptide sequences were predicted to provide 148 peptide class I HLA allele binding combinations. While this selection approach led to an uneven distribution of predicted epitopes from each protein (**Figure 2**), it provided an even distribution of 24 to 27 predicted epitopes for each of the six class I HLA alleles in the panel, with the exception of the HLA-B*4403, which had only 15 predicted epitopes.

### *In Vitro* Analysis of Peptide Binding to HLA Allele Panel

**Figure 3:**
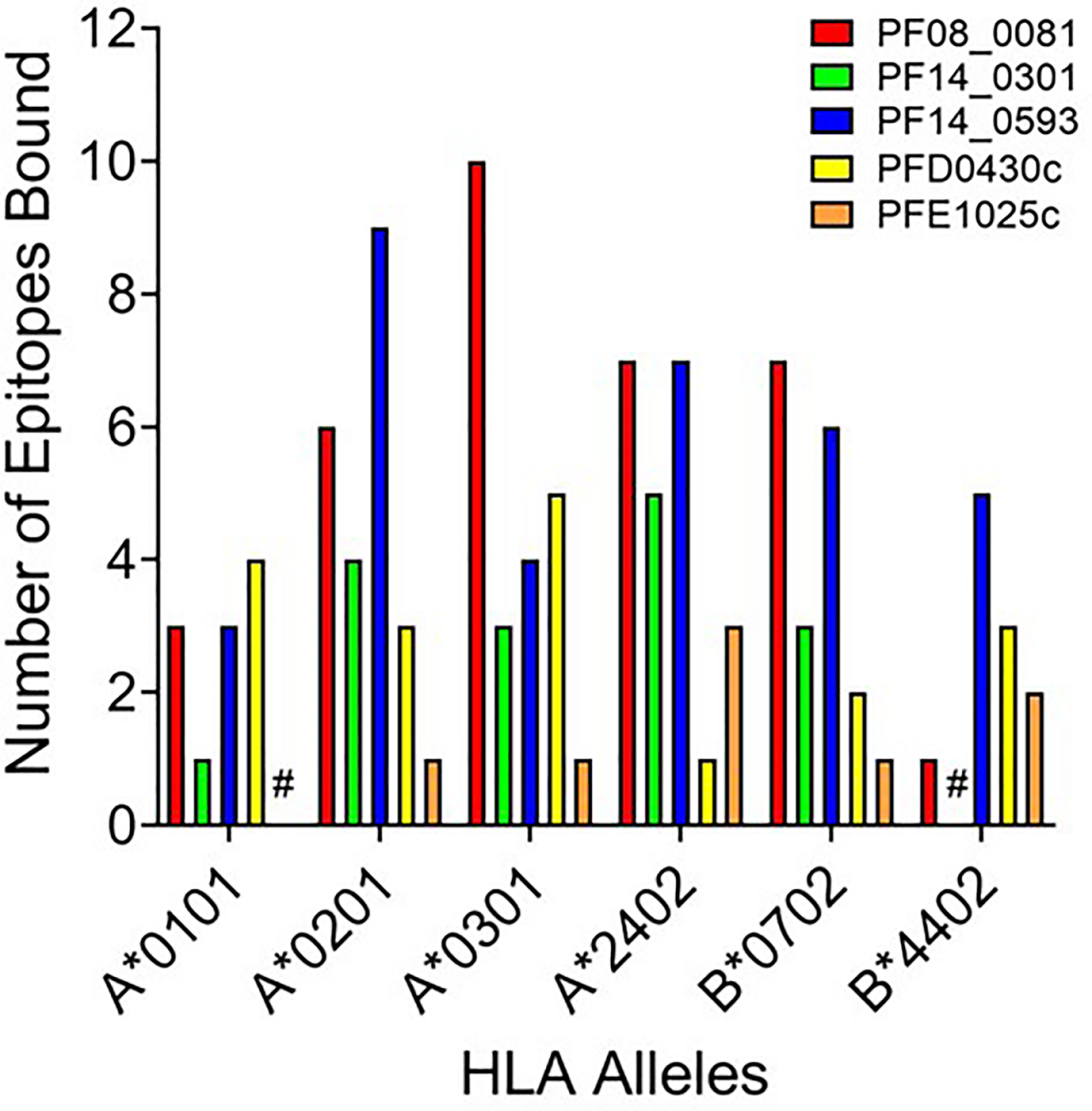
HLA distribution of the number of epitopes for each protein with moderate to high affinity binding to the HLA supertypes. Shown are number of peptides with moderate to high binding affinity to HLA supertype allele for each peptide-allele combinations. Proteins that lacked peptides that bound to a specific class I HLA allele are denoted by #.

While peptide binding to HLA is not an absolute predictor of a peptide’s immunogenicity, high-affinity binding of peptide to cognate HLA can correlate with its ability to elicit an immune response (43–45). To assess the affinity of each peptide for the cognate HLA allele, the selected peptide sequences were synthesized and evaluated for binding using a competition-based *in vitro* HLA binding assay. This assay is based on measuring IC_50_ using non-labeled competitor peptides where the IC_50_ is a representation of binding strength and is proportional to its affinity. Notably, compared to other affinity calculations, the fluorescence polarization method applied here often generates higher IC_50_ concentrations for equivalent peptides. For this reason, IC_50_ thresholds have been adjusted to reflect the affinities most likely to generate a T cell response. Herein, the relative peptide affinity was based on IC_50_ measurements (**Supplementary Table 3**). Experimental peptides were considered very high affinity if they inhibited 50% of control peptide binding at a concentration of 5,000 nM or less, high affinity if they inhibited 50% at a concentration between 5,000 nM and 50,000 nM, and moderate affinity if they inhibited 50% at a concentration between 50,000 nM and 350,000 nM. Experimental peptides were considered low affinity if they inhibited 50% of control peptide binding at concentrations between 350,000 nM and 1,000,000 nM. Experimental peptides that failed to inhibit at least 50% of control peptide binding at any concentration below 1,000,000 nM and do not show a dose response were considered to have negligible affinity and are listed as non-binders (NB) in this analysis. The predicted binding of the peptides was evaluated using the associated HLA supertype allele in all cases except for the predictions for the B*4403 allele, which was not available for analysis. Instead, binding was evaluated using the B*4402 HLA allele. For each peptide-allele combination tested, a color-coded estimate of IC_50_ is presented, with increasing affinity indicated by increasing intensity of blue color (**Supplementary Table 3** **)**. In **Figure 3** the distribution by HLA allele of the peptide-allele combinations that bound with moderate to high affinity for each protein are shown. For the 148 peptide-allele combinations, 110 combinations bound with moderate to high affinity and confirmed a total of 117 predictions. Of note, the concordance between prediction and *in vitro* HLA binding to HLA A*0101 and HLA B*4403 were low and below expectation. Although the binding was tested using the HLA B*4402 allele, the peptides with moderate to high affinity binding for the B*4402 allele aligned well with the higher probability-predictions for the B*4403 allele. Results of the *in vitro* assessments for the selected peptides were such that two of the proteins lacked peptides that bound to two of the class I HLA alleles: PFE1025c lacked peptides binding A*0101 and PF14_0301 lacked peptides binding B*4402 (**Figure 3**). While our analysis did not evaluate the full repertoire of epitopes predicted to bind the six HLA alleles, it did include many of the epitopes from *P. falciparum* proteins found to be conserved in the *P. vivax* orthologue. With the understanding that the proteins used as sources for the peptides will need to be evaluated for protective immunity, we targeted the proteins containing conserved epitopes that covered all six class I alleles. Thus, only the peptides derived from the proteins PF08_0081, PF14_0593, and PFD0430c were advanced for further analysis. A total of sixty-four conserved peptides with an IC_50_ below 100,000 nM were selected from these three proteins for further testing.

### *Ex Vivo* Evaluation of Peptide Immunogenicity

To evaluate whether peptides from PF08_0081, PF14_0593, and PFD0430c induce an immune response, the peptides were tested in the MIMIC^®^ system, a fully autologous human cell-based platform that enables the evaluation of adaptive recall immunity *in vitro*. The MIMIC^®^ system optimizes standard *in vitro* PBMC culture systems *via* recombination of distinct leukocyte components of the immune system at ratios similar to those found at sites of *in vivo* lymphocyte activation. Through this design, the system is able to recapitulate cellular and humoral responses (46). In brief, human PBMC, monocyte-derived DC were pulsed with peptides and co-cultured with naïve CD8 T cells to allow T cell priming. Following a 12-day incubation, primed CD8 T cells were harvested and restimulated with fresh autologous peptide-pulsed DC for 7 hours after which CD8 T cell recall responses were measured by ICCS for IL-2, IFNγ, TNF-α, and granzyme B. The results of the analysis are summarized in **Tables 3**–**5**, which provides a heat map indicating the percentage of donors responding to each peptide for each of the monitored responses. HLA supertype alleles demonstrated to bind the peptide are indicated above each column, and responses aligned to the HLA supertype allele of the PBMC donors are provided in the second column. Five donors were tested for each HLA type, with the exception of the HLA A*02 allele for which six donors were tested (**Supplementary Table 4**). Of the 64 peptides evaluated, 52 demonstrated ≥2-fold increase in IFNγ, TNF-α, and/or granzyme B in at least one donor, with 29 demonstrating a cytokine increase in two or more donors. Notably, IFNγ was the most abundantly expressed cytokine. In general, strong cytokine responses (i.e., responses in 3 or more donors) trended together, with IFNγ and TNF-α increases closely correlated. Granzyme B increases, which are typically associated with a strong immune response, trended less with the IFNγ responses than did TNF-α; however, granzyme B increases did align well with the strongest IFNγ responses. While IL-2 was the second most abundantly expressed cytokine, increases in IL-2 alone were not considered a positive response in the assay. Nevertheless, increases in IL-2 indicate and confirm reactivation of the T cells. Of note, in a few cases (e.g., peptide 131-139 in PF14_0593) IL-2 responses were more dominant than IFNγ or other cytokines assayed. In **Table 6** are epitopes that stimulated ≥2-fold IFNγ response in at least 3 donors and represent the strongest epitopes identified in this study. Taken together these analyses identified fifteen peptides that elicited dominant CD8 T cell responses.

**Table 3:** Summary of immune response to the class I peptides for PF08_0081 by different donor HLA supertypes.

	Allele bound	A*01	A*01, A*03	A*02	A*02	A*02		A*03	A*03	A*03	A*03	A*03	A*03	A*03	A*24	A*24	A*24	A*24	A*24	A*24	B*07	B*07	B*07	B*07	B*44
	Peptide position	380-388	483-491	53-61	70-78	516-524		90-99	101-110	351-359	379-387	391-400	483-492	589-597	5-13	55-63	101-109	387-395	488-496	490-498	67-75	355-363	467-475	520-528	95-104
	Allele tested																								
**IFNγ**	A*01																								
	A*02																								
	A*03																								
	A*24																								
	B*07																								
	B*44																								
**TNFα**	A*01																								
	A*02																								
	A*03																								
	A*24																								
	B*07																								
	B*44																								
**IL-2**	A*01																								
	A*02																								
	A*03																								
	A*24																								
	B*07																								
	B*44																								
**Granzyme B**	A*01																								
	A*02																								
	A*03																								
	A*24																								
	B*07																								
	B*44																								

Individual peptides are identified at the top of the table. The specific cytokine or protease measured is indicated on the left side of the table. The color intensity indicates the number of donors in each HLA type which responded to the peptide and follows: 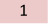

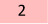

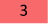

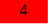

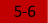
.

**Table 4:** Summary of immune response to the class I peptides for PF14_0593 by different donor HLA supertypes.

	Allele bound	A*01	A*01	A*02	A*02	A*02	A*02	A*02	A*02	A*02	A*03	A*03	A*03	A*03	A*24	A*24, A*02	A*24	A*24	A*24	B*07	B*07	B*07	B*07	B*07	B*44	B*44	B*44	B*44	B*44
	Peptide position	836-844	1091-1099	171-180	307-315	477-485	801-809	863-871	986-994	1138-1146	309-318	315-323	345-354	611-619	490-498	715-723	737-746	738-746	1147-1155	131-139	407-415	625-633	966-974	1256-1264	626-634	645-653	758-766	902-910	1045-1053
	Allele tested																												
**IFNγ**	A*01																												
	A*02																												
	A*03																												
	A*24																												
	B*07																												
	B*44																												
**TNFα**	A*01																												
	A*02																												
	A*03																												
	A*24																												
	B*07																												
	B*44																												
**IL-2**	A*01																												
	A*02																												
	A*03																												
	A*24																												
	B*07																												
	B*44																												
**Granzyme B**	A*01																												
	A*02																												
	A*03																												
	A*24																												
	B*07																												
	B*44																												

Individual peptides are identified at the top of the table. The specific cytokine or protease measured is indicated on the left side of the table. The color intensity indicates the number of donors in each HLA type which responded to the peptide and follows: 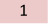

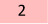

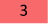

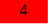

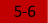
.

**Table 5:** Summary of immune response to the class I peptides for PFD0430c by different donor HLA supertypes.

	Allele bound	A*01	A*01	A*02	A*02, A*24	A*02	A*03	A*03	A*03	A*03	A*03	B*07	B*07	B*44
	Peptide position	237-245	550-558	285-293	371-379	523-531	29-37	72-81	339-347	357-365	570-579	257-265	534-542	440-448
	Allele tested													
**IFNγ**	A*01													
	A*02													
	A*03													
	A*24													
	B*07													
	B*44													
**TNFα**	A*01													
	A*02													
	A*03													
	A*24													
	B*07													
	B*44													
**IL-2**	A*01													
	A*02													
	A*03													
	A*24													
	B*07													
	B*44													
**Granzyme B**	A*01													
	A*02													
	A*03													
	A*24													
	B*07													
	B*44													

Individual peptides are identified at the top of the table. The specific cytokine or protease measured is indicated on the left side of the table. The color intensity indicates the number of donors in each HLA type which responded to the peptide and follows: 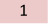

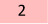

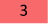

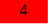

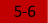
.

**Table 6:** Class I peptides eliciting an immune response in three or more donors.

Protein	PF08_0081						PF14_0593				PFD0430c				
Peptide Position	53-61	55-63	67-75	70-78	490-498	516-524	715-723	986-994	1138-1146	1147-1155	29-37	72-81	237-245	371-379	534-542
Allele															
A*01												x	x		
A*02	x			x		x	x	x	x				x	x	x
A*03											x				
A*24	x	x	x	x	x		x	x		x			x		x
B*07				x											
B*44	x					x	x	x	x				x		

## Discussion

Discovery that sporozoite infection constrained to the liver can confer sterile immunity against malaria led to a focus on identifying liver stage antigens to develop novel subunit vaccines (13). Attenuated sporozoite-induced sterile immunity requires a CD8 T cell response targeting pre-erythrocytic stage antigens (8–12) of which a limited number of antigens targeting the liver stage of malaria have been tested in humans including CSP, TRAP, LSA-1, LSA-3, EXP-1, STARP, and cell-traversal protein for ookinetes and sporozoites (27). Two subunit vaccines targeting the pre-erythrocytic stage of malaria have demonstrated limited protective immunity in humans, RTS,S and ME-TRAP (based on the antigens CSP and TRAP, respectively). Notably, analysis performed in this study indicates that CSP and TRAP are predicted to be poor immunogens (EpiMatrix Protein Scores of -20.66 and -26.46, respectively) and are indicated to contain relatively low numbers of T cell epitopes (**Supplementary Table 1**). For the ME-TRAP vaccine, the T cell epitope content was increased by the addition of a multi-epitope string containing CD8 and CD4 T cell epitopes from TRAP, CSP, EXP-1, LSA-1, LSA-3, and STARP (22). The work present here was undertaken to increase the number of validated T cell epitopes that may be incorporated into future subunit vaccines targeting pre-erythrocytic malaria.

Early attempts to identify T cell epitopes using epitope-driven vaccine design include the development of EP1300 (23). EP1300 is composed of a total of 38 CD8 and 16 CD4 T cell epitopes derived from TRAP, CSP, EXP-1 and LSA-1 expressed as a single protein (23). Many of the epitopes from TRAP and the other proteins included in EP1300 were selected based upon demonstration that the peptides elicited T cell responses in mouse models, including HLA transgenic mice (23). However, when EP1300 was tested in humans, it did not elicit detectable immunity. Whether the lack of response to EP1300 was due to the delivery platform and/or that the selected epitopes were not immunogenic in humans is unclear. In the current study we decided to verify immunogenicity of the screened and selected T cell epitopes using the MIMIC^®^ system to model human T cell immune responses rather than testing in murine models. Notably, this is the first evaluation of liver stage malarial epitopes using the CD8 T cell MIMIC^®^ assay, which has great potential as a tool for early screening of novel T cell malarial antigens. The MIMIC^®^ system uses human DCs and autologous CD8 T cells to model initial T cell priming and subsequent recall of the immune response to the exposed peptides and is scalable to assess responses from a range of human donors. Further, this system allows relatively rapid evaluation of different immune components including cytokines, response elements, and cell markers across a diverse donor cell population (47) where the strength of the analysis is based on the number of donors that respond rather than strictly the magnitude of response. Using the MIMIC^®^ system, we identified several peptides that elicited a CD8 T cell response characterized by an increase in IFNγ, TNF-α, and/or granzyme B with many peptides inducing more than one immune effector. In this regard, it has been shown experimentally that protection is correlated with the induction of T cells expressing IFNγ (8, 16, 48, 49) suggesting IFNγ may contribute to sterile immunity; however, this is unclear in humans.

Using multiple donors for each HLA allele in the panel, the MIMIC^®^ system models the immune response to peptides in a diverse population. Of the sixty-seven peptide-HLA allele combinations demonstrated by the binding analysis and tested in MIMIC^®^, forty-five resulted in at least one donor with the predicted HLA allele responding to the peptide. Further, by selecting peptides that stimulated T cell responses in at least three donors expressing a given HLA allele, we have identified fifteen peptides that stimulate T cell activation across all six of the targeted HLA alleles, representing six distinct supertypes in a larger population (**Table 6**). However, the six peptides stimulating a response to HLA-B*44 should be interpreted cautiously as none were predicted to stimulate cytokine production from this allele. Rather these peptides were predicted to bind HLA-A*02 or HLA-A*01/A*24, which all of the HLA-B*44-matched donors carried. Some class I epitopes have been demonstrated to be promiscuous, with immune activation observed in individuals with HLA alleles that do not match the predicted population of HLA types (50, 51). Thus, the potential promiscuous immune stimulation in the B*44 allele by the peptides listed in **Table 6** cannot be ruled out and warrants further investigation.

While we were able to identify several peptides that elicit an immune response in three or more HLA-matched donors, our results further illustrate the difficulty of predicting class I peptides that stimulate immunity in a larger population (**Supplementary Table 5**). Peptides that bound HLA-A*02 and HLA-A*24 *in vitro* were associated with *ex vivo* HLA-match donor T cell stimulation with a 50% and 33% correlation, respectively. The correlation for the remaining alleles were lower as the correlation of peptide binding to peptides stimulating immunity for HLA-A*01 and HLA-B*44 were 17% and 0%, respectively. This outcome may be due to the relatively low number of available peptides for these alleles as well as the predominantly moderate affinity of the peptides in the binding assay (**Supplementary Table 3**). By contrast, peptides predicted to bind HLA-A*03 and HLA-B*07 demonstrated high affinity binding *in vitro* and had modest correlation to immune stimulation *ex vivo*. For HLA-A*03, seventeen peptides demonstrated binding *in vitro*, fourteen of which bound HLA-A*03 with high affinity; however, only a single peptide from this set stimulated a MIMIC^®^ immune response from at least three HLA-matched donors. Similarly, for HLA-B*07, ten peptides bound this allele *in vitro* yet none stimulated a CD8 T cell response in three or more HLA-matched donors. Taken together, the HLA-A*03 and HLA-B*07 results suggest that the naïve T cell precursor frequency specific for the tested peptides is extremely low in the tested donors, resulting in undetected responses.

The three selected proteins, PF08_0081, PF14_0593, and PFD0430c, have been reported in previous studies. Transcript expression of PF08_0081 and PF14_0593 is increased during *P. yoelii* liver stage infection of mice and in *P. falciparum* infection of human erythrocytes (41, 42, 52, 53). Antibody responses to each of these three proteins have been detected in individuals naturally infected with either *P. falciparum* or *P. vivax* (54–56). However, unlike PF08_0081 and PF14_0593, B cell epitopes specific for PFD0430c have yet to be identified (57), despite that fact that of the three proteins PFD0430c is the best characterized. PFD0430c was first identified by transcriptome analysis of *P. yoelii* salivary gland sporozoites, and was located in the microneme (58). Its expression is essential for sporozoite penetration of the liver sinusoidal cell layer, an early step in establishing liver infection (59, 60). In volunteers vaccinated with irradiated sporozoites, PFD0430c-specific T cell responses are elicited (39). Experimentally, the *P. yoelii* orthologue of PFD0430c was included in a minigene library of eighty-nine proteins used to discover protective *Plasmodium* pre-erythrocytic T cells antigens (61). Unfortunately, the results were inclusive due to the overall lack of T cell responses to the proteins, including the PDF0430c orthologue, and the fact that the CSP domain used as a control did not result in the expected high frequency of T cell activation.

The EpiVax in silico analysis predicted many more potential epitopes in the top 5% of Z-scores for each of the class I HLA alleles across the selected proteins (**Table 2**), although most of those were not conserved. With less than 10% of the predicted epitopes tested, it is likely each protein contains additional effective epitopes that were not evaluated in this study. In conclusion, the data demonstrate the successful iterative application of in silico predictions to identify class I peptides that bind to predicted HLA alleles and stimulate human T cells *ex vivo*. These results lay the foundation for future work to evaluate whether the peptides identified here are naturally presented by *P. falciparum* infected liver cells and determine the potential of each peptide to stimulate protective immunity in malaria vaccination.

## Data Availability Statement

The original contributions presented in the study are included in the article/**Supplementary Material**. Further inquiries can be directed to the corresponding author.

## Ethics Statement

The studies involving human participants were reviewed and approved by Advarra, Protocol CRRI 0906009. The patients/participants provided their written informed consent to participate in this study.

## Author Contributions

Overall conceptualization and study designs were contributed by AN, KT, TP, VK, and GG. In silico analyses and *in vitro* HLA binding assays were performed by FT, LM, and PH, with supervision by WM and AG. *Ex vivo* lymphocyte activation assays were performed and formally analyzed by BS and ES, with supervision by DD. Project management, data organization and formal analyses were performed by KT and AN. Manuscript writing was performed by AN, KT, TP, and BS. Manuscript editing and review were performed by GG, VK, FT, LM, AG, and BS. All authors contributed to the article and approved the submitted version.

## Funding

These studies were made possible through support provided by the Office of Infectious Diseases, Bureau for Global Health, U.S. Agency for International Development (https://www.usaid.gov), under the terms of the Malaria Vaccine Development Program (MVDP) Contract AID-OAA-C-15-00071, for which Leidos, Inc. is the prime contractor. The opinions expressed herein are those of the authors and do not necessarily reflect the views of the U.S. Agency for International Development. The funders approved study plans but had no direct role in development of study designs, data collection/analysis, or preparation of the manuscript.

## Conflict of Interest

AN, TP, KT, VK, and GG are employees of Leidos, Inc., the prime contractor for Malaria Vaccine Development Program (MVDP) Contract AID-OAA-C-15-00071. FT, LM, WM, and AG are employees of EpiVax, Inc., an MVDP subcontractor. BS, ES, and DD are employees of Sanofi Pasteur, an MVDP subcontractor. BS, DD, and ES hold Sanofi shares and/or stock options. PH was a previous employee of EpiVax, Inc.

The remaining authors declare that the research was conducted in the absence of any commercial or financial relationships that could be construed as a potential conflict of interest.
