# Supplementary material for: Identification, Selection and Immune Assessment of Liver Stage CD8 T Cell Epitopes From Plasmodium falciparum
Source: Front Immunol. 2021 May 7;12:684116. doi: 10.3389/fimmu.2021.684116 (PMC8138313; doi:10.3389/fimmu.2021.684116)
Supplement: Supplementary file 4 [file Table_4.docx]

Table S4 HLA profile of PBMC donors

| **Donor** | **HLA-A** | **HLA-B** |
| --- | --- | --- |
| 1594 | A*01:AUUHE A*24:AUUHF | B*07:AUUSE B*57:AUUVE |
| 3648 | A*02:AGHMX A*29:AGGDM | B*44:AUUVA B*49:AUUVB |
| 4461 | A*02:AUUHH A*24:AUUHJ | B*44:AUUVR B*50:AUUVS |
| 5004 | A*01:AUUHE A*24:AUUHF | B*44:AUUUN B*57:AUUUP |
| 5457 | A*03:AUUHC A*03:AUUHC | B*07:AUVMU B*07:AUVMV |
| 5589 | A*24:AUUHB A*68:ANMVB | B*35:05 B*52:AUUUG |
| 6304 | A*02:AGHMX A*24:AUUBD | B*35:12 B*50:AUUSH |
| 6348 | A*01:AUUAM A*03:AUUAY | B*07:AUUSE B*58:AUUSG |
| 6369 | A*01:AUUAG A*03:AUUAH | B*08:AUUPM B*14:AUUPN |
| 6637 | A*02:AUTWS A*02:ANZKZ | B*40:CFGU B*44:AUURP |
| 6914 | A*01:AUUNK A*02:AUUNM | B*08:AUUVN B*44:AUUSK |
| 8762 | A*03:AUSZG A*31:AUSZP | B*07:AUUWD B*51:AUUNZ |
| 8805 | A*02:AVPPP A*03:AVPPR | B*07:AVPKU B*51:AVPKV |
