# Supplementary material for: Identification, Selection and Immune Assessment of Liver Stage CD8 T Cell Epitopes From Plasmodium falciparum
Source: Front Immunol. 2021 May 7;12:684116. doi: 10.3389/fimmu.2021.684116 (PMC8138313; doi:10.3389/fimmu.2021.684116)
Supplement: Supplementary file 5 [file Table_5.docx]

Table S5 Number of peptides stimulating donor CD8 T cells with HLA supertype alleles matching the predicted binding HLA allele

|  | **A*0101** | **A*0201** | **A*0301** | **A*2402** | **B*0702** | **B*4403** |
| --- | --- | --- | --- | --- | --- | --- |
| Number of Peptides Predicted to Stimulate Immunity | 6 | 14 | 17 | 12 | 11 | 7 |
| Number Immune Responses in One or More Donors | 4 | 13 | 8 | 8 | 7 | 5 |
| Number of Immune Responses in Three or More Donors | 1 | 7 | 1 | 4 | 0 | 0 |
